# Supplementary material for: Seminoma and Embryonal Carcinoma Footprints Identified by Analysis of Integrated Genome-Wide Epigenetic and Expression Profiles of Germ Cell Cancer Cell Lines
Source: PLoS One. 2014 Jun 2;9(6):e98330. doi: 10.1371/journal.pone.0098330 (PMC4041891; doi:10.1371/journal.pone.0098330)
Supplement: File S1 — ZIP file containing DMRforPairs output for significant regions. Please start from the html files. (ZIP) [file pone.0098330.s008.zip › figures/38289.pdf]

RegionID: 38289, chr7:27195918−27196555−M_values

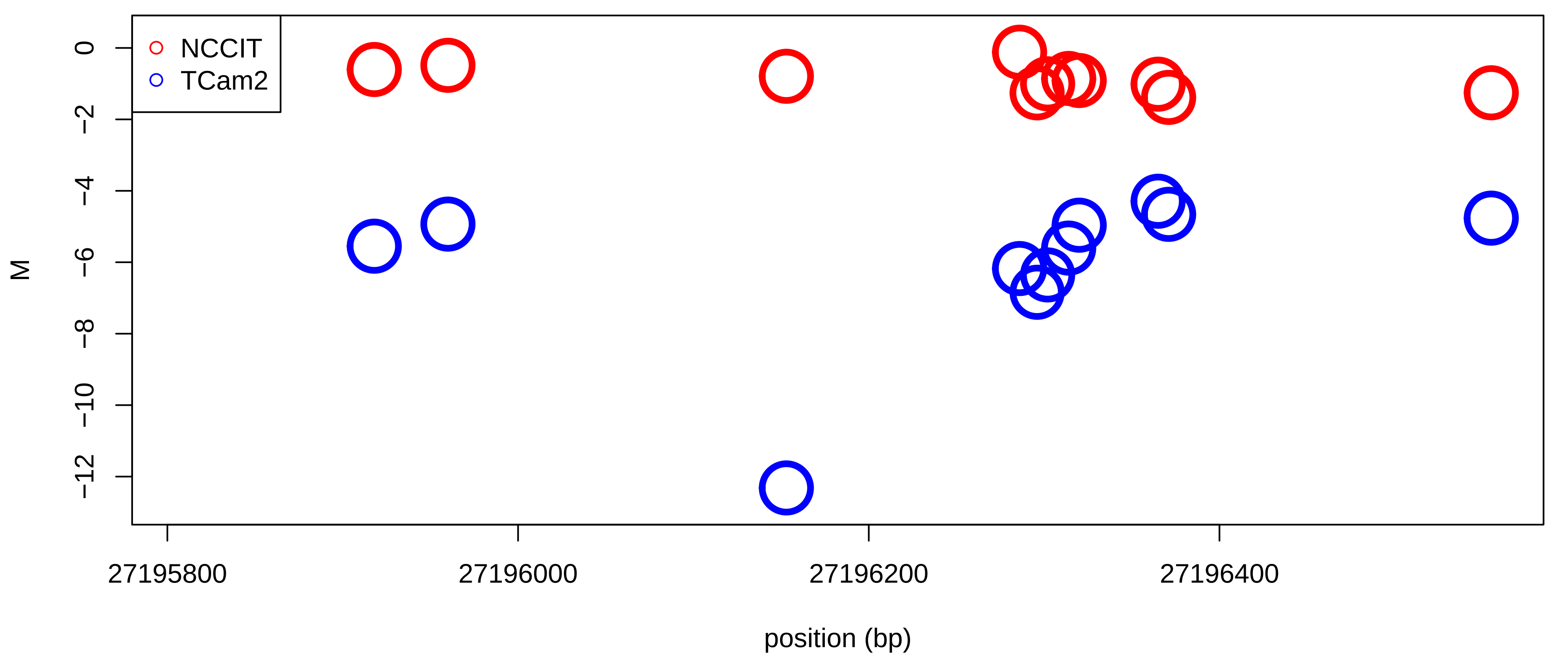

RegionID: 38289, chr7:27195918−27196555−Beta_values

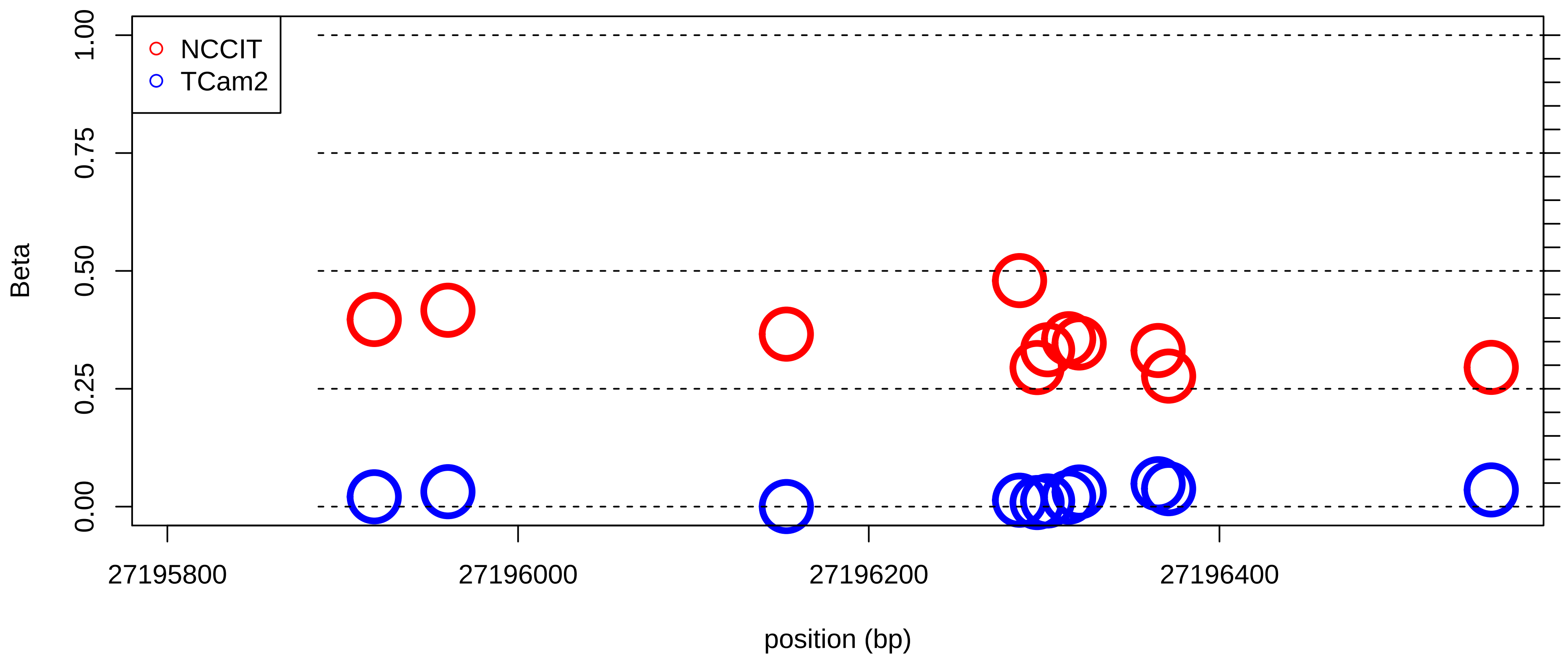

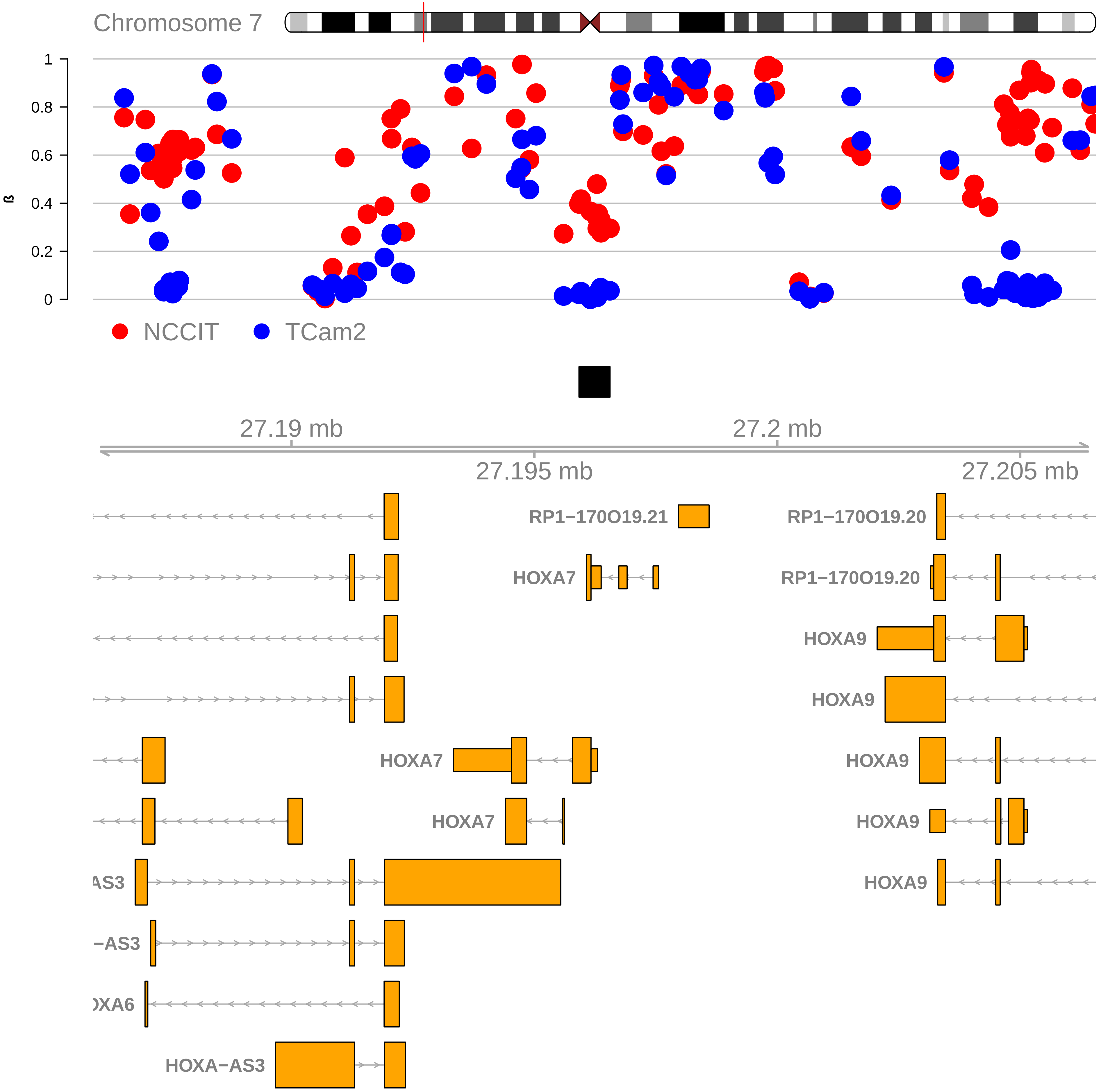

Chromosome 7
ß
1
0.8
0.6
0.4
0.2
0
NCCIT
TCam2
27.19 mb
27.2 mb
27.195 mb
27.205 mb
RP1−170O19.21
RP1−170O19.20
HOXA7
RP1−170O19.20
HOXA9
HOXA9
HOXA7
HOXA9
HOXA7
HOXA9
HOXA9
HOXA−AS3
